# Supplementary material for: Spontaneous symmetry breaking of dissipative optical solitons in a two-component Kerr resonator
Source: Nat Commun. 2021 Jun 29;12:4023. doi: 10.1038/s41467-021-24251-0 (PMC8242005; doi:10.1038/s41467-021-24251-0)
Supplement: Supplementary file 1 — Supplementary information [file 41467_2021_24251_MOESM1_ESM.pdf]

Supplementary information for "Spontaneous symmetry breaking of dissipative optical solitons in a two-component Kerr resonator"

Gang Xu[1,2], Alexander Nielsen[1,2], Bruno Garbin[1,2,3], Lewis Hill[4,5], Gian-Luca Oppo[4], Julien Fatome[1,2,6], Stuart G. Murdoch[1,2], Stéphane Coen[1,2], and Miro Erkintalo[1,2*]
[1] *Department of Physics, University of Auckland, Auckland 1010, New Zealand*
[2] *The Dodd-Walls Centre for Photonic and Quantum Technologies, New Zealand*
[3] *Centre de Nanosciences et de Nanotechnologies (C2N), CNRS, Université Paris-Saclay, F-91120 Palaiseau, France*
[4] *SUPA and Department of Physics, University of Strathclyde, Glasgow G4 0NG, Scotland, EU*
[5] *National Physical Laboratory, Hampton Road, Teddington, TW11 0LW, UK and*
[6] *ICB, UMR 6303 CNRS, Université Bourgogne-Franche-Comté, 9 Av. Alain Savary, BP 47870, F-21078 Dijon, France*

This documents contains five Supplementary Notes and seven Supplementary Figures that provide additional information regarding results presented in the manuscript entitled "*Spontaneous symmetry breaking of dissipative optical solitons in a two-component Kerr resonator*". In particular, the document presents results from additional simulations, theoretical analyses and experiments that further elucidate the physics of the soliton symmetry breaking phenomenon reported in the main manuscript mentioned above.

SUPPLEMENTARY NOTE 1: PARAMETER-DEPENDENCE OF SOLITON SYMMETRY BREAKING

In our main manuscript, we consider the soliton symmetry breaking phenomenon for two different driving power levels $X = 4.5$ and $X = 21$ and for a single cross-coupling coefficient $B = 1.6$. As noted in our manuscript, the phenomenon is not restricted to any specific set of parameters but can rather occur for all cross-coupling coefficients $B > 1$ provided that the driving power X exceeds a given threshold. Moreover, we also note in our manuscript that the ratio of the soliton modal intensities increases with the driving power, corroborating our claim by comparing results for $X = 4.5$ and $X = 21$. Here we further explore the parameter-dependence of the soliton symmetry breaking, providing stronger theoretical evidence to support our statements.

We first consider the soliton symmetry breaking for different values of driving powers X but for a constant cross-coupling coefficient $B = 1.6$. To this end, we computed the soliton bifurcation curves for a range of X using the same methodology as was used to obtain Fig. 2(a) of our main manuscript. Supplementary Fig. 1 shows illustrative examples with X ranging from $X = 5$ to $X = 3$. We see clearly how the "bubble" describing the soliton's asymmetric modal intensities shrinks as the driving power X decreases, ultimately disappearing altogether when $X \lesssim 3.14$. Accordingly, we can identify $X_\mathrm{t} = 3.14$ as the threshold driving power above which the soliton symmetry breaking can occur.

As noted in our main manuscript, the threshold X_t depends on the cross-coupling coefficient B. To explore the shape of the dependency, we repeated the above bifurcation calculations for a range of B values and extracted the threshold power X_t. Results are shown in Supplementary Fig. 2(a), with solid circles highlighting the obtained values $X_\mathrm{t}(B)$. As can be seen, the symmetry breaking threshold decreases with increasing B. Also noted in our manuscript was the fact that the contrast between the solitons' modal intensities increases with the driving power [this trend is also evident in Supplementary Fig. 1]. To gain more insights, we calculated the soliton bifurcation curves for a large range of driving powers and cross-coupling coefficients, and extracted the maximum modal intensity contrast attained. Supplementary Fig. 2(b) shows results from our calculations. We may clearly see how the intensity ratios increase with the driving power and the cross-coupling coefficient, with contrasts exceeding 20 dB expected for $B \approx 2$ and $X \approx 15$. Whilst a detailed discussion is beyond the scope of our present work, we note here that, when the driving power (and/or the cross-coupling coefficient B) exceeds a certain value, the soliton bifurcation structure can become more complicated than those shown in Supplementary Fig. 1. A full investigation onto the rich bifurcations that can occur will be reported elsewhere.

SUPPLEMENTARY NOTE 2: ADDITIONAL RESULTS FOR $X = 21$

In our main manuscript, numerical simulation results were presented only for one of the two driving power levels used in our experiments (Fig. 1, $X = 4.5$). For the sake of completeness, Supplementary Fig. 3 below shows simulation results for the other driving power level used in our experiments ($X = 21$). The results were obtained by using Eqs. (1) of our main manuscript to simulate the intracavity field envelopes $E_1(t,\tau)$ and $E_2(t,\tau)$ as the

* m.erkintalo@auckland.ac.nz

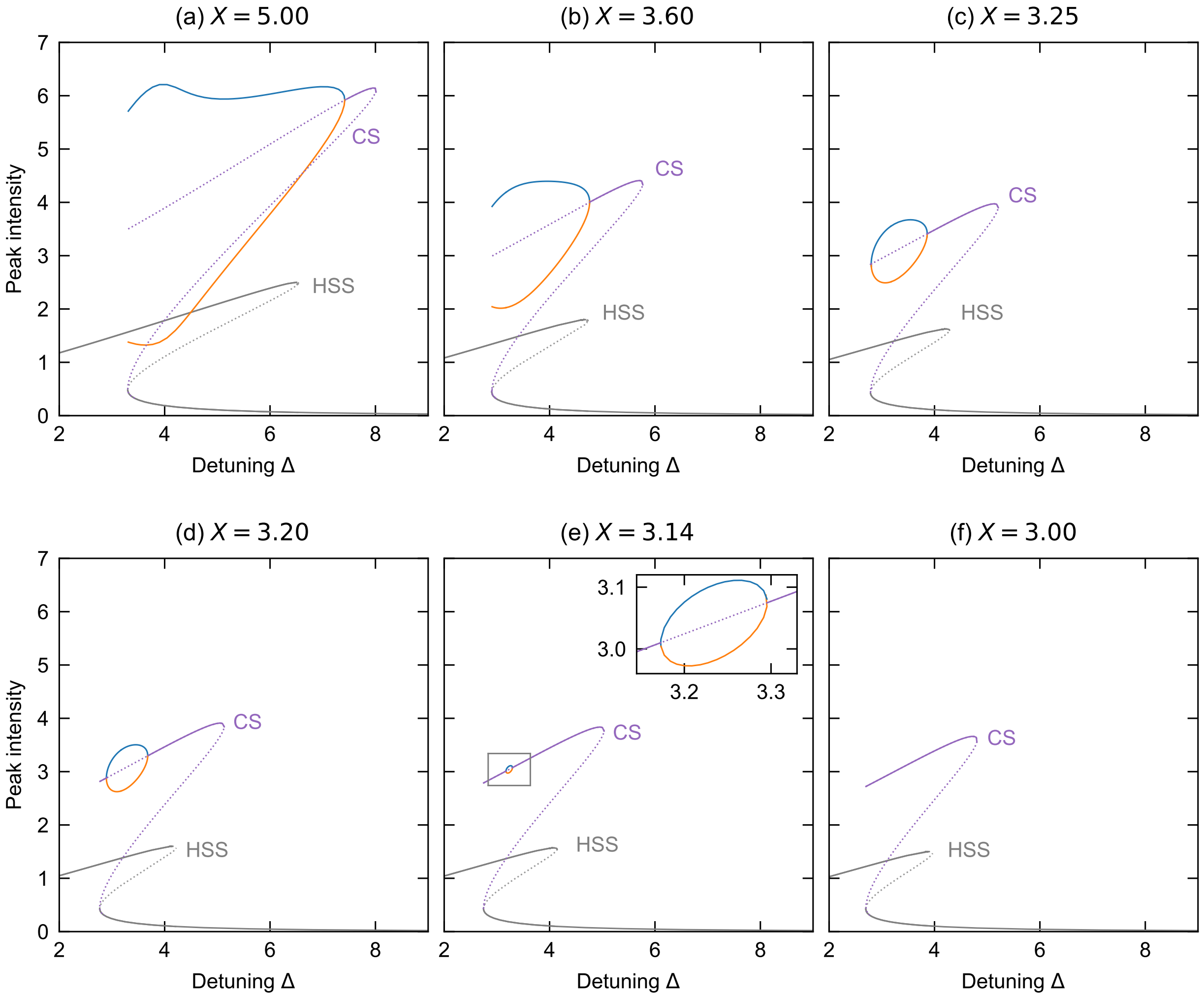

Supplementary Fig. 1. Bifurcation curves illustrating the parameter-dependence of soliton symmetry breaking. (a)–(f) Cavity soliton (CS) and homogeneous steady-state (HSS) bifurcation curves computed as in Fig. 2(a) of our main manuscript for a constant cross-coupling coefficient $B = 1.6$ but a range of driving powers X as indicated. Solid (dashed) curves correspond to stable (unstable) solutions. Inset in (e) shows a zoom around the "bubble" that describes the soliton symmetry breaking.

common cavity detuning Δ was continuously increased, thus mimicking the experimental procedure of scanning the frequency of the driving laser over a cavity resonance [see e.g. Fig. 4(a) of our main manuscript]. Supplementary Fig. 3(a) plots the modal energies as the detuning is scanned over a cavity resonance, showing that, in agreement with corresponding experiments [Fig. 4(a) of our main manuscript], the energies split when the detuning reaches the "soliton step" region. We must of course emphasize that, as noted in our main manuscript, the precise dynamics depend on the detuning scan rate as well as the noise that is present. In particular, the noise-sensitivity of the symmetry-breaking process implies that simulations with different noise conditions (but otherwise identical parameters) will yield different results, in line with the fact that each experimental scan over a cavity resonance yields different intensity traces.

Interestingly, the simulation results in Supplementary Fig. 3(a) reveal that, right after the detuning reaches the "soliton step" region, the symmetry broken CSs that emerge in fact exhibit oscillatory (breathing) behaviour. It is only once the detuning increases further that the oscillations cease and the solitons become stable. The soliton oscillations are evident in the simulated evolution of the modal energy [Supplementary Fig. 3(a)] but cannot be resolved in our experiments [Fig. 4(a) of our main manuscript] due to the slow response of the detector used: with a response time of about 250 round trips, the detector smooths out the oscillations that occur with a period of about 10 round trips. The experimental results shown in Fig. 4(b)–(g) of our main manuscript were obtained with the detuning stabilized at $\Delta \approx 11.5$, where the CSs are stable and do not oscillate (either in simulation or experiment). However, if the detuning is stabilized at a smaller value, our experiments remarkably do reveal the existence of symmetry-broken, breathing CSs. Illustra-

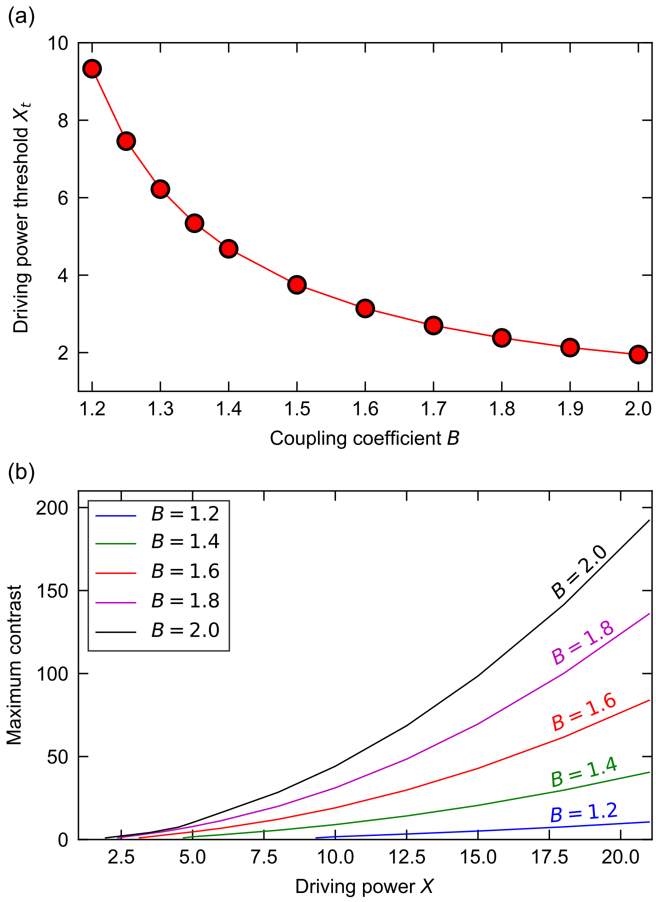

Supplementary Fig. 2. Characteristics of CS symmetry breaking. (a) Solid circles show theoretically predicted threshold driving power $X_{\rm t}$, above which CS symmetry breaking may occur, as a function of the cross-coupling coefficient B. Solid curve is a guide to the eye. (b) Maximum contrast (across different detunings) between the modal intensities of symmetry-broken CSs for a range of driving powers X and cross-coupling coefficients B.

tive measurements are shown in Supplementary Fig. 4. Here the detuning was stabilized at $\Delta \approx 10.5$, where our simulations predict that breathing solitons should manifest themselves; the experiments indeed show clearly how the CSs exhibit persistent and periodic oscillations. To the best of our knowledge, these results constitute the first observations of symmetry-broken, breathing dissipative solitons. It is noteworthy that the modal oscillations observed in Supplementary Fig. 4 occur in-phase, with both amplitudes reaching their maxima (and minima) simultaneously. More extensive calculations show that more complicated dynamics are also possible; a detailed study of such effects will be reported elsewhere.

SUPPLEMENTARY NOTE 3: VECTORIAL PROPERTIES OF SYMMETRY-BROKEN CAVITY SOLITONS

The symmetry-broken cavity solitons (CSs) explored in our work are vectorial in the sense that they display a non-trivial state of polarization that is associated with both of the system's cavity modes. In particular, the fact that the background on top of which the solitons sit exhibits *symmetric* intensity distribution along the two polarization modes implies that, for *asymmetric* solitons, there must be a polarization excursion in the fast time domain. This point is illustrated in Supplementary Fig. 5. Here we consider a single steady-state, symmetry-broken (E_1 dominates) CS structure with intensity and phase profiles as shown in Supplementary Fig. 5(a) and (b), respectively (see figure caption for parameters). In Supplementary Figs. 5(c) and (d), we respectively show the ratio of the modal amplitudes, $|E_2(\tau)|/|E_1(\tau)|$, as well as the fast-time-dependent Stokes parameters

$$S_1(\tau) = 2\mathrm{Re}[E_1 E_2^*], \tag{S1}$$
$$S_2(\tau) = -2\mathrm{Im}[E_1 E_2^*], \tag{S2}$$
$$S_3(\tau) = |E_1|^2 - |E_2|^2, \tag{S3}$$

normalized to the total intensity $S_0(\tau) = |E_1(\tau)|^2 + |E_2(\tau)|^2$. As can be seen, the state of polarization changes continuously across the soliton, connecting to the symmetric polarization of the background field, which is constant in (fast) time and equal to the state of polarization of the driving field. Since the solitons' state of polarization clearly changes as a function of fast time, it cannot be unambiguously associated with either one of the cavity modes (or a simple linear combination thereof). Moreover, we must emphasize that removal of one of the vectorial components of the symmetry-broken CS causes the soliton to either disappear altogether or to regenerate the removed component. Taken together, these features further confirm that the symmetry-broken CSs are genuine vectorial structures, rather than (linear) superpositions of two polarization multiplexed CSs sharing the same velocity.

SUPPLEMENTARY NOTE 4: SWITCHING OF SYMMETRY-BROKEN CAVITY SOLITONS WITH $X = 4.5$

Figure 4 of our main manuscript demonstrates deterministic switching between the two symmetry-broken soliton states. These results were obtained for a driving power $X = 21$, but we again emphasise that the salient phenomenon is not tied to any particular parameters. To illustrate this point, Supplementary Figs. 6(a)–(c) shows experimentally measured switching for $X = 4.5$. Clearly the behaviour is qualitatively similar to the behaviour reported for $X = 21$ in our main manuscript. This be-

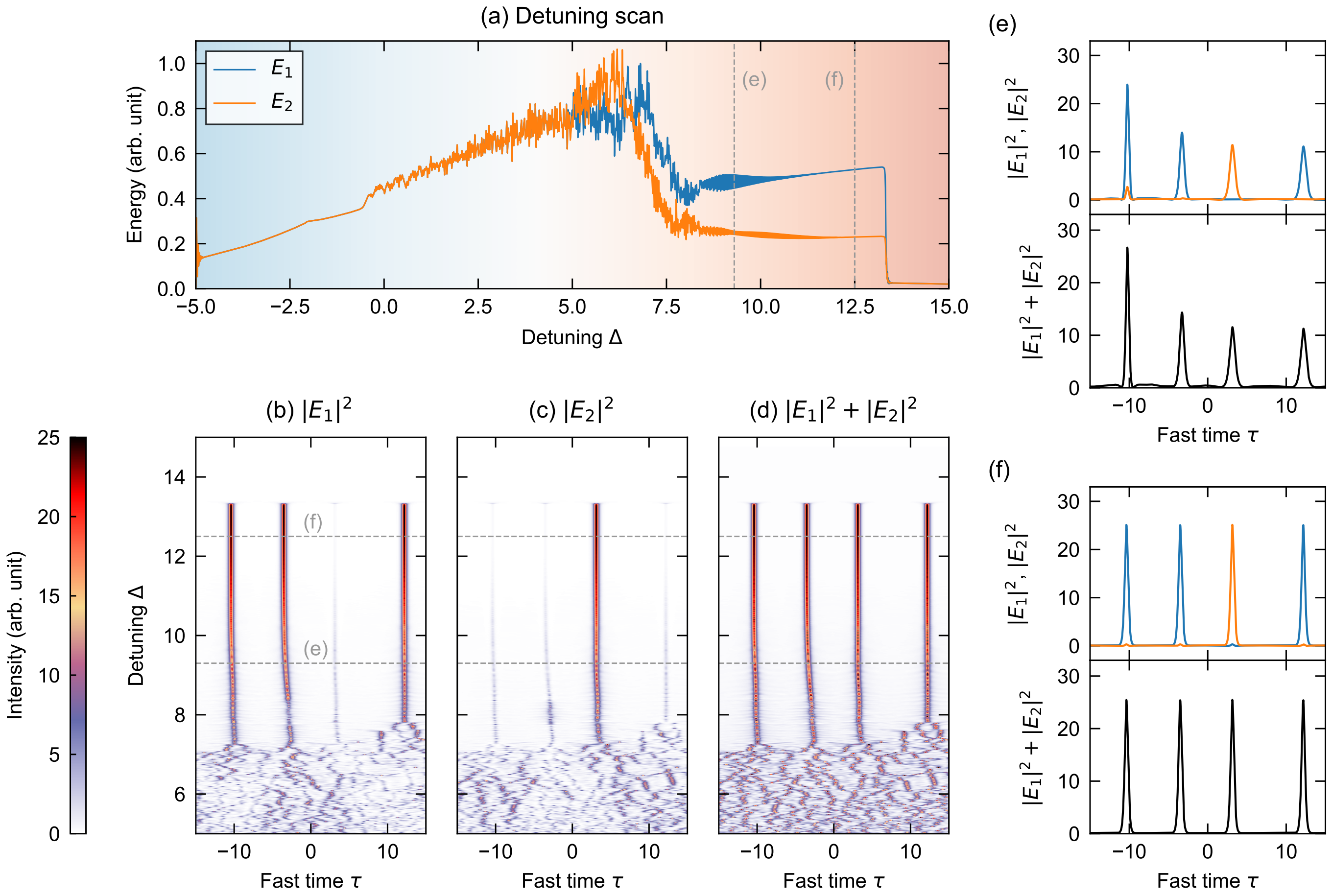

Supplementary Fig. 3. Numerical simulation results, showing the evolution of the intracavity waveform as the detuning Δ is adiabatically scanned for a constant driving power $X = 21$ and cross-coupling coefficient $B = 1.6$. (a) Evolution of the integrated intracavity energy contained in cavity mode E_1 (blue curve) and E_2 (orange curve) as the detuning Δ is linearly increased. (b)–(c) Space-time diagrams showing the evolution of the modal intensities along the (b) E_1 and the (c) E_2 modes; (d) shows the spatiotemporal evolution of the total intensity. (e), (f) Snapshots at selected detunings as indicated, with (e) corresponding to an oscillating and (f) a stable CS regime.

haviour is also readily reproduced by numerical simulations of Eqs. (1) of our main manuscript. Indeed, Supplementary Figs. 6(d)–(f) show results from direct numerical integration of Eqs. (1) of our main manuscript with an initial condition corresponding to a single symmetry-broken CS that is initially aligned predominantly along the E_2 mode. (The integration results were brought into dimensional units by applying the usual transformations of variables [1].) At round trip 1500 a transient polarization perturbation is applied on the driving field, inciting the soliton to permanently switch its polarization to the mirror-image of the initial condition. These dynamics are in very good agreement with our experiments. Note, however, that the simulations capture the true picosecond-scale duration of the CSs whereas the experiments are subject to the finite ~ 80 ps response time of our detection system.

SUPPLEMENTARY NOTE 5: IMPACT OF ASYMMETRIES

Our work focuses on the regime where the two-component system displays perfect symmetry, with the cavity detunings $\Delta_{1,2}$ and driving amplitudes $F_{1,2}$ equal across the two modes $E_{1,2}$. As shown in our work, despite such perfect symmetry, asymmetric CS states can emerge due to spontaneous symmetry breaking (SSB). In our experiments, we carefully adjust the system parameters ($\Delta_{1,2}$ and $F_{1,2}$) to be as symmetric as possible by meticulously looking for the splitting of the modal intensities when scanning over a resonance [e.g. Fig. 3(a) and 4(a) of our manuscript]. Our results clearly demonstrate the existence of symmetry-broken CS, but it is intuitively clear that some degree of asymmetries must be present in our experiments (as is the case for all experiments involving truly spontaneous symmetry breaking). It is therefore interesting to consider how residual asymmetries may impact on the co-existence of the symmetry-

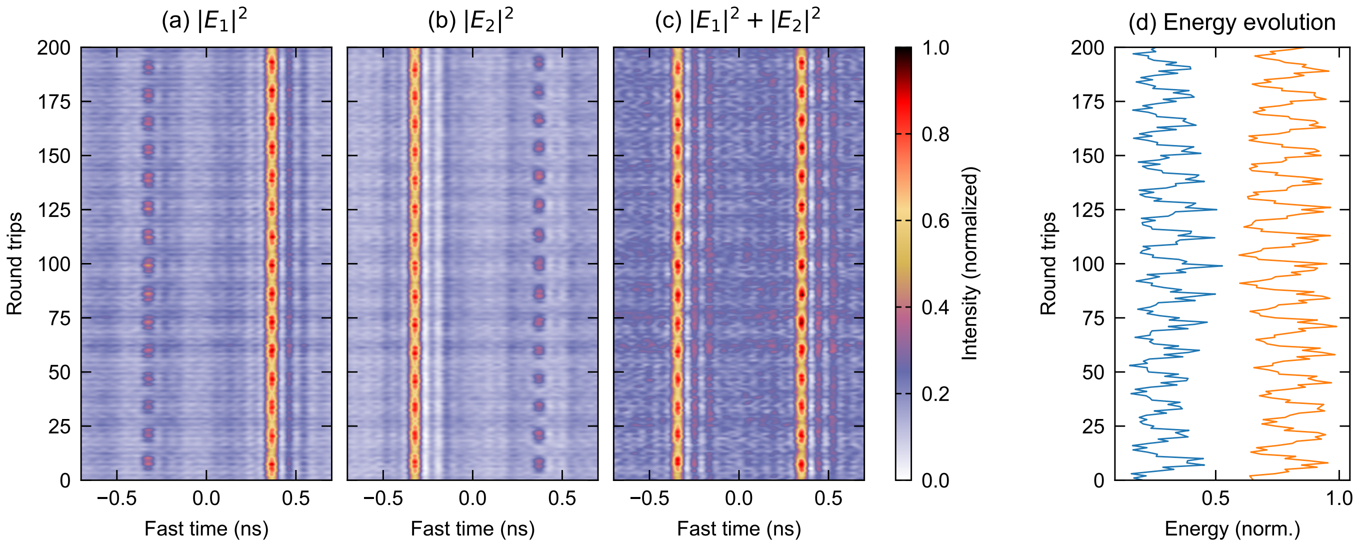

Supplementary Fig. 4. Experimental observation of breathing, symmetry-broken CSs, obtained for a driving power $X = 21$ with the detuning stabilized at $\Delta \approx 10.5$. (a) and (b) are measured space-time diagrams that show the evolution of the modal intensities of two co-existing CS states with different polarization, whilst (c) shows the corresponding evolution of the total intensity. (d) Evolution of the energies along modes E_1 (blue curve) and E_2 (orange curve) for one of the CSs (centred at about -0.4 ns).

broken soliton states.

The impact of asymmetries has been previously studied for the SSB of the homogeneous states of a driven Kerr resonator [2]. Here we extend the theoretical methodology used in ref. [2] to examine the impact of asymmetries on CS symmetry breaking. Specifically, we use the Newton-Raphson method to find the steady-state CS solutions of Eqs. (1) of our manuscript in the presence of asymmetries in the driving field intensities and cavity detunings. For these calculations, we used a fixed total driving intensity $X = 4.5$ (as in Figs. 1–3 of our manuscript) and mean detuning $\Delta_\mathrm{m} = (\Delta_2 + \Delta_1)/2 = 3.9$. Supplementary Fig. 7 shows results from our computations, with Supplementary Figs. 7(a) and 7(b) showing the impact of asymmetric driving intensities and cavity detunings, respectively. Here, the driving field ellipticity angle defines the modal driving amplitudes viz. $F_1 = \sqrt{X}\cos\chi$ and $F_2 = \sqrt{X}\sin\chi$, whilst the difference in wave number $\delta\Delta = \Delta_2 - \Delta_1$, where $\Delta_2 = \Delta_\mathrm{m} + \delta\Delta/2$ and $\Delta_1 = \Delta_\mathrm{m} - \delta\Delta/2$.

As can be seen, the coexistence of two asymmetric soliton states is possible over finite ranges of asymmetries. Whilst these ranges are not very large, they are readily accessible with our experiments. Of course, as is characteristic to SSB phenomena, fully random selection between the two different asymmetric states occurs only in the absence of any asymmetry (such that the pitchfork is whole). In the presence of asymmetries, one of the states dominates over the other, and is accessed with a higher probability when a control parameter (e.g. cavity detuning) is swept across the bifurcation point. In our experiments, we optimise the system's symmetry by looking for the regime where the two soliton states are accessed with equal probability.

[1] F. Leo, S. Coen, P. Kockaert, S.-P. Gorza, P. Emplit, and M. Haelterman, "Temporal cavity solitons in one-dimensional Kerr media as bits in an all-optical buffer," Nature Photon. **4**, 471–476 (2010).

[2] B. Garbin, J. Fatome, G.-L. Oppo, M. Erkintalo, S. G. Murdoch, and S. Coen, "Asymmetric balance in symmetry breaking," Phys. Rev. Research **2**, 023244 (2020).

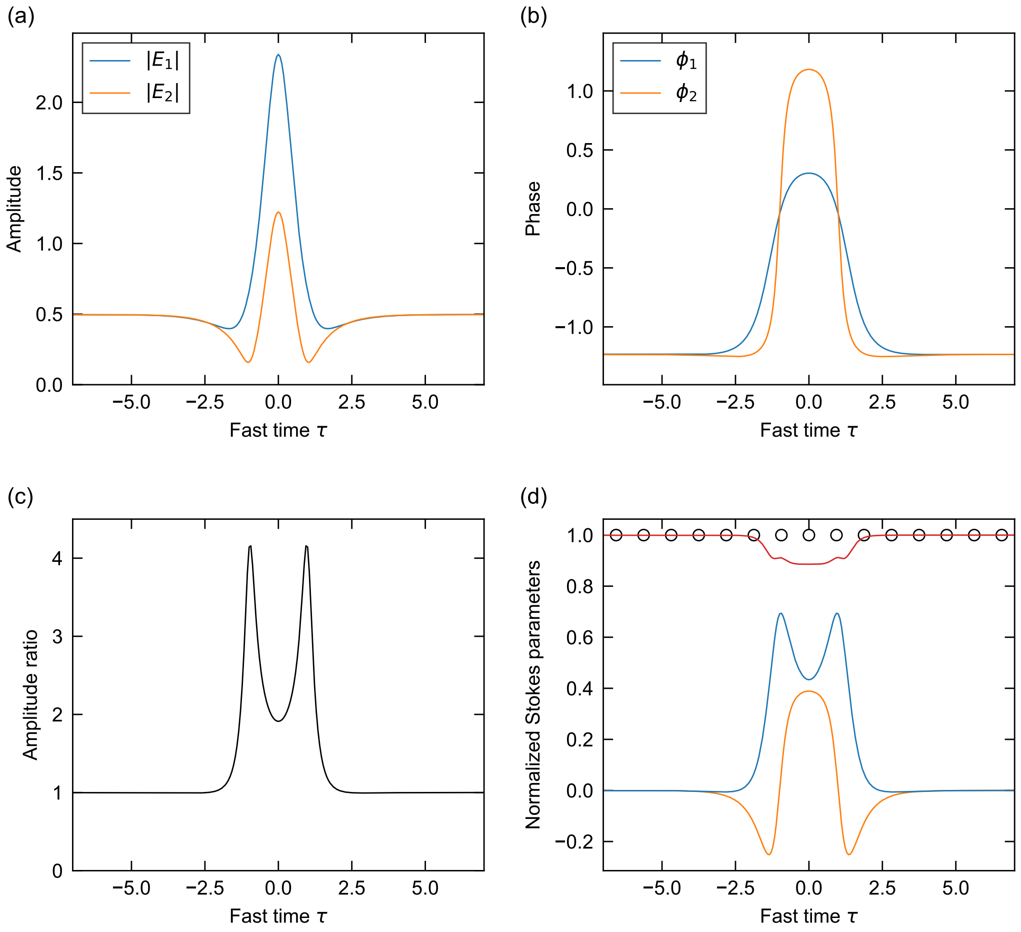

Supplementary Fig. 5. Polarization characteristics of symmetry-broken CSs. (a) Amplitude and (b) phase profiles of a steady-state, symmetry-broken CS that exists for $B = 1.6$, $X = 4.5$, and $\Delta = 3.5$. The CS was obtained as the steady-state solution of Eqs. (1) of our main manuscript using the Newton-Raphson method. (c) Ratio of the modal amplitudes, $|E_2(\tau)|/|E_1(\tau)|$, corresponding to data shown in (a). (d) Normalized Stokes parameters $S_1(\tau)/S_0(\tau)$ (red curve), $S_2(\tau)/S_0(\tau)$ (orange curve), and $S_3(\tau)/S_0(\tau)$ (blue curve) for the CS waveform shown in (a) and (b). Also shown as black open circles in (d) is the normalized Stokes parameter $S_1(\tau)/S_0(\tau) = 1$ for the driving field.

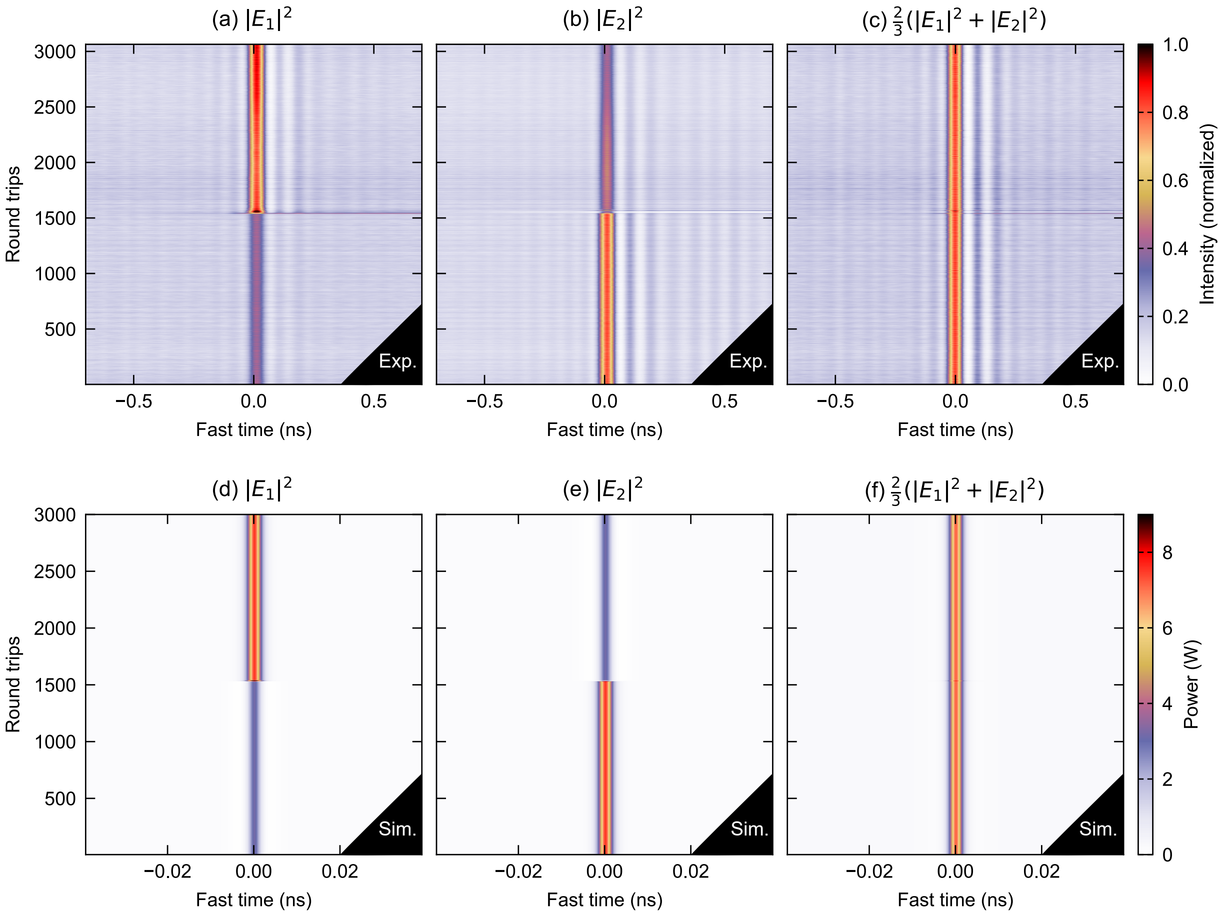

Supplementary Fig. 6. Deterministic switching of symmetry-broken CSs for $X = 4.5$. (a)–(c) Experimentally measured space-time diagrams obtained for $X = 4.5$, demonstrating polarization switching of a symmetry-broken CS. (a) E_1 mode; (b) E_2 mode; (c) total intensity. A polarization perturbation is applied on the driving field at round trip 1500. (d)–(f) Numerical simulation results corresponding to experimental data in (a)–(c), respectively. Note that data in panels (c) and (f) is scaled by 2/3 such that the top and bottom colorbars apply to (a)–(c) and (d)–(f), respectively.

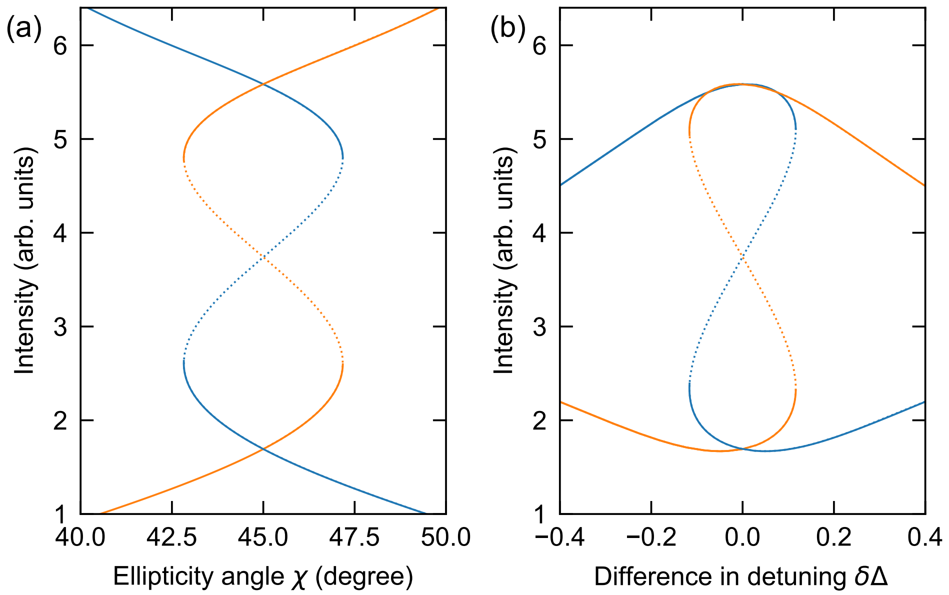

Supplementary Fig. 7. Results from theoretical calculations that show the impact of asymmetries on the symmetry-broken CS states. (a) and (b) show the peak modal intensities of the symmetry-broken CS states as a function of the (a) driving field ellipticity χ and (b) difference in cavity detunings $\delta\Delta = \Delta_2 - \Delta_1$. Blue and orange curves correspond to intensities along the E_1 and E_2 modes, respectively, with solid (dotted) curves corresponding to stable (unstable) solutions. The calculations consider a constant total intensity $X = 4.5$ and mean detuning $\Delta_\mathrm{m} = (\Delta_2 + \Delta_1)/2 = 3.9$.
